# 14-(2,3-Dichloro­phen­yl)-9,10-dimethyl­benzimidazo[1,2-*a*]benzo[*f*][1,8]naphthyridine-6-carbonitrile

**DOI:** 10.1107/S1600536809008447

**Published:** 2009-03-19

**Authors:** Andrii V. Tarasov, Tatyana A. Volovnenko, Roman I. Zubatyuk, Oleg V. Shishkin, Yulian M. Volovenko

**Affiliations:** a National Taras Shevchenko University, Department of Chemistry, 64 Volodymyrska Str., Kyiv 01601, Ukraine; b STC "Institute for Single Crystals", National Academy of Science of Ukraine, 60 Lenina Ave., Kharkiv 61001, Ukraine

## Abstract

In the title compound, C_27_H_16_Cl_2_N_4_, the benzimidazo[1,2-*a*]benzo[*f*][1,8]naphthyridine system is nearly planar (r.m.s. deviation for all non-H atoms = 0.033 Å). The dichloro­phenyl substituent is rotated by −67.5 (2)° from this plane. In the crystal structure, mol­ecules form stacks along the crystallographic (100) direction due to π–π stacking inter­actions with a centroid–centroid distance of 3.4283 (9) Å.

## Related literature

For the synthesis of the title compound and a series of similar compounds, see: Volovnenko *et al.* (2006 ▶). For 1,2-fused benzimidazo heterocycles and their fluorescence properties, see: Gokhale & Seshadri (1987 ▶); Rajagopal & Seshadri (1991 ▶). For the biological properties of isoquinoline derivatives, see: Shamma (1972 ▶); Kametami & Fukomoto (1981 ▶); Bijan & Basu (1965 ▶); Neumeyer & Weinhard (1970 ▶).
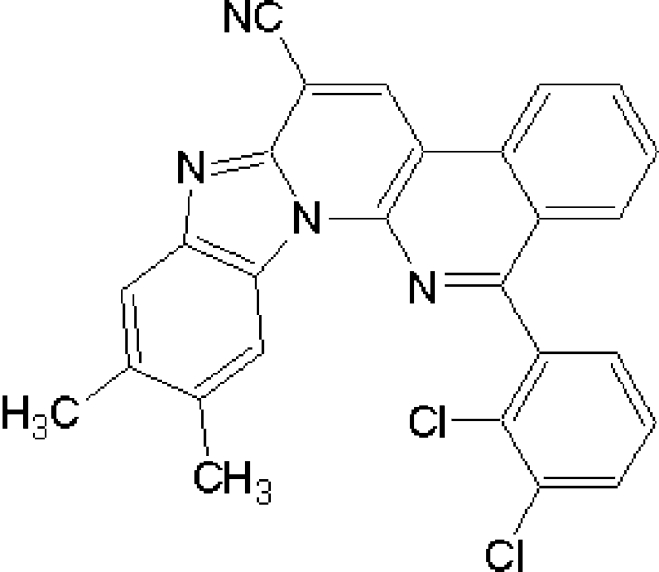


## Experimental

### 

#### Crystal data

C_27_H_16_Cl_2_N_4_
*M* *_r_* = 467.34
Triclinic, 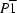

*a* = 8.5588 (8) Å
*b* = 11.0751 (13) Å
*c* = 12.2332 (11) Å
α = 76.985 (9)°
β = 75.986 (8)°
γ = 85.438 (9)°
*V* = 1095.77 (19) Å^3^
*Z* = 2
Mo *K*α radiation
μ = 0.32 mm^−1^
*T* = 293 K
0.6 × 0.1 × 0.1 mm

#### Data collection

Oxford-Diffraction Xcalibur-3 diffractometer
Absorption correction: multi-scan (*CrysAlis RED*; Oxford Diffraction, 2009 ▶) *T*_min_ = 0.82, *T*_max_ = 0.97
20237 measured reflections
4290 independent reflections
2405 reflections with *I* > 2σ(*I*)
*R*_int_ = 0.034

#### Refinement

*R*[*F* ^2^ > 2σ(*F* ^2^)] = 0.034
*wR*(*F* ^2^) = 0.072
*S* = 1.01
4290 reflections
300 parameters
H-atom parameters constrained
Δρ_max_ = 0.19 e Å^−3^
Δρ_min_ = −0.24 e Å^−3^

### 

Data collection: *CrysAlis Pro* (Oxford Diffraction, 2009 ▶); cell refinement: *CrysAlis Pro*; data reduction: *CrysAlis Pro*; program(s) used to solve structure: *SHELXS97* (Sheldrick, 2008 ▶); program(s) used to refine structure: *SHELXL97* (Sheldrick, 2008 ▶); molecular graphics: *ORTEP-3 for Windows* (Farrugia, 1997 ▶) and *Mercury* (Macrae *et al.*, 2006 ▶); software used to prepare material for publication: *publCIF* (Westrip, 2009 ▶).

## Supplementary Material

Crystal structure: contains datablocks I, global. DOI: 10.1107/S1600536809008447/sj2589sup1.cif

Structure factors: contains datablocks I. DOI: 10.1107/S1600536809008447/sj2589Isup2.hkl

Additional supplementary materials:  crystallographic information; 3D view; checkCIF report

## Figures and Tables

**Fig. 1.**
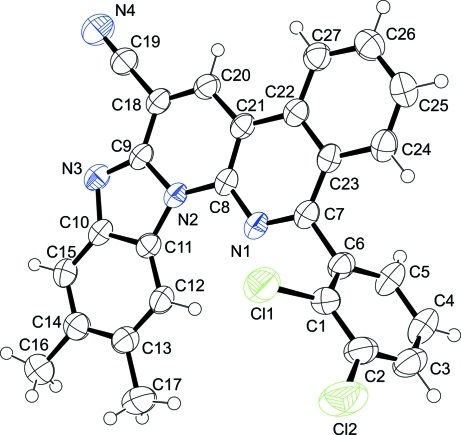
The molecular structure of the title compound, with atom labels and 50% probability displacement ellipsoids for non-H atoms.

**Fig. 2.**
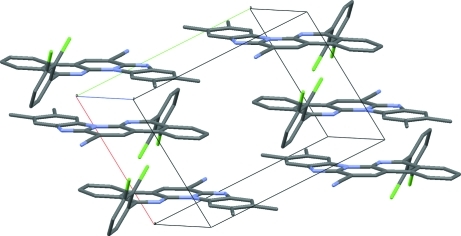
Crystal packing of the title compound showing stacks along the a axis. Hydrogen atoms are not shown.

## supplementary crystallographic information

### Comment

In the past few decades 1,2-fused benzimidazo heterocycles have attracted attention because of their fluorescent properties (Gokhale & Seshadri, 1987; Rajagopal & Seshadri 1991). On the other hand isoquinoline derivatives exhibit a wide range of biological effects and are of great interest to synthetic as well as pharmaceutical organic chemists (Shamma, 1972; Kametami & Fukomoto, 1981; Bijan & Basu, 1965; Neumeyer & Weinhard, 1970).

In a previous paper (Volovnenko *et al.*, 2006) we have described the synthesis of a series of 14-arylbenzimidazo[1,2-*a*]benzo[*f*]-1,8-naphthyridine-6-carbonitriles. We report herein the crystal structure of the title compound, which is the derivative of the new heterocyclic system, benzimidazo[1,2-*a*]benzo[*f*]-1,8-naphthyridine.

The molecular structure of the title compound is illustrated in Fig. 1. The benzimidazo[1,2-*a*]benzo[*f*]-1,8-naphthyridine system is nearly planar (RMS deviation of the non-hydrogen atoms from mean plane is 0.033 Å). Benzene ring is rotated with respect to this plane (the C5—C6—C7—C23 torsion angle is -67.5 (2)°). This rotation results in the loss of conjugation between π systems of the heterocycle and benzene ring. In crystal molecules form stacked chains along the *a* axis (Fig. 2) due to stacking interactions between the π systems of the pentacyclic fragments. The distance between the parallel planes is 3.4186 (8) Å.

### Experimental

The title compound was synthesized by the reaction of 3-chloro-1-(2,3-dichlorophenyl)isoquinoline-4-carbaldehyde (1 mmol) with (5,6-dimethyl-1*H*-benzimidazol-2-yl)acetonitrile (1 mmol) in dimethylformamide (3–4 ml). After refluxing for 3 h, the reaction mixture was left to stand for overnight. The resulting crude solid was filtered, washed twice with acetone (10 ml) and dried. Yield: 65%. Crystals suitable for X-ray analysis were obtained by slow crystallization from hot dimethylformamide.

### Refinement

H-atoms were placed in calculated positions with d(C—H)=0.93–0.96 Å and refined using riding model with *U*_iso_(H) = n*U*_eq_(C) (n = 1.2 for aromatic C—H and n = 1.5 for methyl groups).

### Crystal data

**Table:**

C_27_H_16_Cl_2_N_4_	*Z* = 2
*M**_r_* = 467.34	*F*(000) = 480
Triclinic, *P*1	*D*_x_ = 1.416 Mg m^−^^3^
Hall symbol: -P 1	Mo *K*α radiation, λ = 0.71069 Å
*a* = 8.5588 (8) Å	Cell parameters from 6294 reflections
*b* = 11.0751 (13) Å	θ = 2.7–28.6°
*c* = 12.2332 (11) Å	µ = 0.32 mm^−^^1^
α = 76.985 (9)°	*T* = 293 K
β = 75.986 (8)°	Prism, pale orange
γ = 85.438 (9)°	0.6 × 0.1 × 0.1 mm
*V* = 1095.77 (19) Å^3^	

### Data collection

**Table:**

Oxford-Diffraction Xcalibur-3 diffractometer	4290 independent reflections
Radiation source: Enhance (Mo) X-ray Source	2405 reflections with *I* > 2σ(*I*)
graphite	*R*_int_ = 0.034
Detector resolution: 16.1827 pixels mm^-1^	θ_max_ = 26.0°, θ_min_ = 2.7°
ω scans	*h* = −10→10
Absorption correction: multi-scan (*CrysAlis RED*; Oxford Diffraction, 2009)	*k* = −13→13
*T*_min_ = 0.82, *T*_max_ = 0.97	*l* = −15→15
20237 measured reflections	

### Refinement

**Table:**

Refinement on *F*^2^	Primary atom site location: structure-invariant direct methods
Least-squares matrix: full	Secondary atom site location: difference Fourier map
*R*[*F*^2^ > 2σ(*F*^2^)] = 0.034	Hydrogen site location: inferred from neighbouring sites
*wR*(*F*^2^) = 0.072	H-atom parameters constrained
*S* = 1.01	*w* = 1/[σ^2^(*F*_o_^2^) + (0.03*P*)^2^] where *P* = (*F*_o_^2^ + 2*F*_c_^2^)/3
4290 reflections	(Δ/σ)_max_ < 0.001
300 parameters	Δρ_max_ = 0.19 e Å^−^^3^
0 restraints	Δρ_min_ = −0.24 e Å^−^^3^

### Special details

**Table:**

Geometry. All e.s.d.'s (except the e.s.d. in the dihedral angle between two l.s. planes) are estimated using the full covariance matrix. The cell e.s.d.'s are taken into account individually in the estimation of e.s.d.'s in distances, angles and torsion angles; correlations between e.s.d.'s in cell parameters are only used when they are defined by crystal symmetry. An approximate (isotropic) treatment of cell e.s.d.'s is used for estimating e.s.d.'s involving l.s. planes.
Refinement. Refinement of *F*^2^ against ALL reflections. The weighted *R*-factor *wR* and goodness of fit *S* are based on *F*^2^, conventional *R*-factors *R* are based on *F*, with *F* set to zero for negative *F*^2^. The threshold expression of *F*^2^ > σ(*F*^2^) is used only for calculating *R*-factors(gt) *etc*. and is not relevant to the choice of reflections for refinement. *R*-factors based on *F*^2^ are statistically about twice as large as those based on *F*, and *R*- factors based on ALL data will be even larger.

### Fractional atomic coordinates and isotropic or equivalent isotropic displacement parameters (Å^2^)

**Table:**

	*x*	*y*	*z*	*U*_iso_*/*U*_eq_	
Cl1	0.53369 (8)	0.08996 (5)	0.11087 (4)	0.07512 (19)	
Cl2	0.55737 (9)	0.26410 (6)	−0.13175 (4)	0.0980 (2)	
N1	0.24410 (16)	0.07841 (12)	0.33502 (10)	0.0416 (4)	
N2	0.15186 (16)	−0.10485 (12)	0.46081 (10)	0.0380 (3)	
N3	0.05348 (17)	−0.29056 (13)	0.56580 (11)	0.0445 (4)	
N4	0.1445 (2)	−0.31290 (16)	0.85388 (14)	0.0799 (6)	
C1	0.4249 (2)	0.22794 (16)	0.09828 (14)	0.0478 (5)	
C2	0.4347 (2)	0.30496 (19)	−0.01113 (14)	0.0553 (5)	
C3	0.3485 (2)	0.41496 (19)	−0.02191 (16)	0.0599 (6)	
H3A	0.3550	0.4660	−0.0944	0.072*	
C4	0.2526 (2)	0.45022 (18)	0.07372 (17)	0.0637 (6)	
H4A	0.1933	0.5244	0.0653	0.076*	
C5	0.2435 (2)	0.37670 (17)	0.18209 (15)	0.0575 (5)	
H5A	0.1802	0.4023	0.2465	0.069*	
C6	0.3291 (2)	0.26398 (16)	0.19503 (13)	0.0439 (4)	
C7	0.3168 (2)	0.18469 (15)	0.31350 (13)	0.0411 (4)	
C8	0.22786 (19)	0.00654 (15)	0.44182 (13)	0.0373 (4)	
C9	0.1249 (2)	−0.19032 (15)	0.56664 (13)	0.0388 (4)	
C10	0.0316 (2)	−0.27288 (15)	0.45439 (13)	0.0402 (4)	
C11	0.0918 (2)	−0.15919 (15)	0.38690 (13)	0.0388 (4)	
C12	0.0814 (2)	−0.12061 (16)	0.27243 (14)	0.0469 (5)	
H12A	0.1221	−0.0450	0.2283	0.056*	
C13	0.0089 (2)	−0.19800 (17)	0.22635 (14)	0.0506 (5)	
C14	−0.0526 (2)	−0.31292 (16)	0.29292 (15)	0.0471 (5)	
C15	−0.0407 (2)	−0.34975 (16)	0.40614 (14)	0.0457 (4)	
H15A	−0.0808	−0.4256	0.4501	0.055*	
C16	−0.1334 (2)	−0.39560 (17)	0.24225 (15)	0.0624 (6)	
H16A	−0.1458	−0.4763	0.2922	0.094*	
H16B	−0.0686	−0.4025	0.1679	0.094*	
H16C	−0.2374	−0.3607	0.2343	0.094*	
C17	−0.0053 (3)	−0.1566 (2)	0.10197 (16)	0.0844 (7)	
H17A	0.0436	−0.0775	0.0691	0.127*	
H17B	−0.1170	−0.1497	0.0995	0.127*	
H17C	0.0485	−0.2164	0.0588	0.127*	
C18	0.1780 (2)	−0.15844 (16)	0.65802 (13)	0.0421 (4)	
C19	0.1568 (2)	−0.24516 (17)	0.76740 (15)	0.0527 (5)	
C20	0.2535 (2)	−0.05020 (16)	0.64046 (13)	0.0431 (4)	
H20A	0.2882	−0.0312	0.7009	0.052*	
C21	0.28103 (19)	0.03485 (15)	0.53223 (13)	0.0375 (4)	
C22	0.36455 (19)	0.14875 (15)	0.50764 (13)	0.0392 (4)	
C23	0.3824 (2)	0.22500 (15)	0.39544 (13)	0.0396 (4)	
C24	0.4691 (2)	0.33565 (16)	0.36838 (15)	0.0491 (5)	
H24A	0.4811	0.3866	0.2954	0.059*	
C25	0.5350 (2)	0.36845 (17)	0.44770 (15)	0.0534 (5)	
H25A	0.5927	0.4409	0.4284	0.064*	
C26	0.5162 (2)	0.29336 (17)	0.55808 (16)	0.0530 (5)	
H26A	0.5612	0.3166	0.6120	0.064*	
C27	0.4328 (2)	0.18652 (16)	0.58793 (14)	0.0462 (5)	
H27A	0.4208	0.1381	0.6620	0.055*	

### Atomic displacement parameters (Å^2^)

**Table:**

	*U*^11^	*U*^22^	*U*^33^	*U*^12^	*U*^13^	*U*^23^
Cl1	0.1038 (5)	0.0659 (4)	0.0530 (3)	0.0237 (3)	−0.0178 (3)	−0.0166 (3)
Cl2	0.1300 (6)	0.1142 (5)	0.0412 (3)	0.0138 (5)	−0.0102 (3)	−0.0149 (3)
N1	0.0466 (9)	0.0348 (8)	0.0384 (8)	−0.0005 (7)	−0.0089 (7)	0.0011 (7)
N2	0.0416 (9)	0.0340 (8)	0.0337 (8)	0.0004 (7)	−0.0077 (6)	0.0009 (6)
N3	0.0464 (9)	0.0416 (9)	0.0402 (9)	−0.0028 (8)	−0.0085 (7)	0.0013 (7)
N4	0.1199 (17)	0.0661 (12)	0.0471 (10)	−0.0184 (12)	−0.0227 (10)	0.0103 (9)
C1	0.0549 (13)	0.0468 (11)	0.0441 (11)	−0.0044 (10)	−0.0180 (9)	−0.0066 (9)
C2	0.0628 (14)	0.0632 (13)	0.0401 (11)	−0.0086 (11)	−0.0163 (10)	−0.0043 (10)
C3	0.0633 (15)	0.0666 (15)	0.0446 (12)	−0.0157 (12)	−0.0172 (11)	0.0085 (10)
C4	0.0627 (15)	0.0506 (12)	0.0649 (14)	0.0010 (11)	−0.0148 (11)	0.0131 (10)
C5	0.0586 (14)	0.0491 (12)	0.0512 (12)	0.0007 (10)	−0.0083 (10)	0.0114 (10)
C6	0.0466 (12)	0.0427 (11)	0.0392 (10)	−0.0091 (9)	−0.0111 (9)	0.0019 (8)
C7	0.0421 (11)	0.0370 (10)	0.0383 (10)	0.0007 (9)	−0.0047 (8)	−0.0015 (8)
C8	0.0360 (10)	0.0325 (10)	0.0373 (10)	0.0022 (8)	−0.0036 (8)	−0.0011 (8)
C9	0.0392 (11)	0.0362 (10)	0.0341 (10)	0.0024 (9)	−0.0040 (8)	0.0012 (8)
C10	0.0399 (11)	0.0368 (10)	0.0381 (10)	0.0028 (9)	−0.0071 (8)	0.0004 (8)
C11	0.0380 (11)	0.0387 (10)	0.0375 (10)	0.0033 (8)	−0.0085 (8)	−0.0054 (8)
C12	0.0576 (13)	0.0360 (10)	0.0430 (11)	−0.0020 (9)	−0.0112 (9)	−0.0003 (9)
C13	0.0597 (13)	0.0474 (12)	0.0431 (11)	0.0017 (10)	−0.0130 (10)	−0.0062 (9)
C14	0.0433 (11)	0.0476 (11)	0.0505 (11)	0.0035 (9)	−0.0107 (9)	−0.0125 (9)
C15	0.0464 (12)	0.0358 (10)	0.0512 (11)	−0.0033 (9)	−0.0095 (9)	−0.0027 (9)
C16	0.0685 (15)	0.0579 (13)	0.0648 (13)	−0.0059 (11)	−0.0189 (11)	−0.0160 (10)
C17	0.133 (2)	0.0714 (15)	0.0532 (13)	−0.0218 (15)	−0.0388 (14)	0.0033 (11)
C18	0.0460 (12)	0.0394 (11)	0.0356 (10)	0.0031 (9)	−0.0074 (8)	−0.0010 (8)
C19	0.0657 (14)	0.0483 (12)	0.0418 (11)	−0.0077 (10)	−0.0122 (10)	−0.0025 (10)
C20	0.0480 (12)	0.0437 (11)	0.0365 (10)	0.0034 (9)	−0.0109 (8)	−0.0064 (8)
C21	0.0366 (11)	0.0365 (10)	0.0357 (9)	0.0051 (8)	−0.0062 (8)	−0.0041 (8)
C22	0.0352 (11)	0.0384 (10)	0.0408 (10)	0.0061 (9)	−0.0049 (8)	−0.0083 (8)
C23	0.0396 (11)	0.0338 (10)	0.0401 (10)	0.0024 (8)	−0.0035 (8)	−0.0043 (8)
C24	0.0539 (13)	0.0398 (11)	0.0470 (11)	−0.0029 (10)	−0.0032 (9)	−0.0041 (9)
C25	0.0542 (13)	0.0448 (11)	0.0598 (13)	−0.0071 (10)	−0.0039 (10)	−0.0161 (10)
C26	0.0537 (13)	0.0525 (12)	0.0561 (12)	0.0000 (10)	−0.0118 (10)	−0.0195 (10)
C27	0.0501 (12)	0.0441 (11)	0.0436 (10)	0.0043 (10)	−0.0108 (9)	−0.0094 (9)

### Geometric parameters (Å, °)

**Table:**

Cl1—C1	1.7214 (18)	C12—H12A	0.9300
Cl2—C2	1.7201 (19)	C13—C14	1.411 (2)
N1—C7	1.319 (2)	C13—C17	1.519 (2)
N1—C8	1.3495 (19)	C14—C15	1.379 (2)
N2—C8	1.3873 (19)	C14—C16	1.505 (2)
N2—C11	1.4009 (19)	C15—H15A	0.9300
N2—C9	1.4013 (19)	C16—H16A	0.9600
N3—C9	1.3123 (19)	C16—H16B	0.9600
N3—C10	1.3886 (19)	C16—H16C	0.9600
N4—C19	1.138 (2)	C17—H17A	0.9600
C1—C6	1.387 (2)	C17—H17B	0.9600
C1—C2	1.403 (2)	C17—H17C	0.9600
C2—C3	1.370 (3)	C18—C20	1.357 (2)
C3—C4	1.373 (2)	C18—C19	1.441 (2)
C3—H3A	0.9300	C20—C21	1.421 (2)
C4—C5	1.378 (2)	C20—H20A	0.9300
C4—H4A	0.9300	C21—C22	1.436 (2)
C5—C6	1.393 (2)	C22—C27	1.408 (2)
C5—H5A	0.9300	C22—C23	1.420 (2)
C6—C7	1.500 (2)	C23—C24	1.416 (2)
C7—C23	1.426 (2)	C24—C25	1.360 (2)
C8—C21	1.397 (2)	C24—H24A	0.9300
C9—C18	1.425 (2)	C25—C26	1.397 (2)
C10—C15	1.394 (2)	C25—H25A	0.9300
C10—C11	1.401 (2)	C26—C27	1.363 (2)
C11—C12	1.392 (2)	C26—H26A	0.9300
C12—C13	1.382 (2)	C27—H27A	0.9300
			
C7—N1—C8	117.45 (14)	C15—C14—C16	119.31 (16)
C8—N2—C11	131.25 (13)	C13—C14—C16	120.72 (16)
C8—N2—C9	123.19 (13)	C14—C15—C10	119.72 (16)
C11—N2—C9	105.54 (13)	C14—C15—H15A	120.1
C9—N3—C10	104.64 (13)	C10—C15—H15A	120.1
C6—C1—C2	119.87 (16)	C14—C16—H16A	109.5
C6—C1—Cl1	120.71 (13)	C14—C16—H16B	109.5
C2—C1—Cl1	119.42 (14)	H16A—C16—H16B	109.5
C3—C2—C1	119.80 (17)	C14—C16—H16C	109.5
C3—C2—Cl2	119.59 (15)	H16A—C16—H16C	109.5
C1—C2—Cl2	120.60 (15)	H16B—C16—H16C	109.5
C2—C3—C4	120.37 (17)	C13—C17—H17A	109.5
C2—C3—H3A	119.8	C13—C17—H17B	109.5
C4—C3—H3A	119.8	H17A—C17—H17B	109.5
C3—C4—C5	120.64 (18)	C13—C17—H17C	109.5
C3—C4—H4A	119.7	H17A—C17—H17C	109.5
C5—C4—H4A	119.7	H17B—C17—H17C	109.5
C4—C5—C6	119.98 (18)	C20—C18—C9	120.27 (14)
C4—C5—H5A	120.0	C20—C18—C19	120.64 (15)
C6—C5—H5A	120.0	C9—C18—C19	119.04 (15)
C1—C6—C5	119.33 (15)	N4—C19—C18	178.0 (2)
C1—C6—C7	121.37 (15)	C18—C20—C21	121.74 (15)
C5—C6—C7	119.29 (15)	C18—C20—H20A	119.1
N1—C7—C23	123.58 (14)	C21—C20—H20A	119.1
N1—C7—C6	116.15 (14)	C8—C21—C20	119.06 (15)
C23—C7—C6	120.27 (15)	C8—C21—C22	116.76 (14)
N1—C8—N2	115.72 (14)	C20—C21—C22	124.16 (14)
N1—C8—C21	125.72 (15)	C27—C22—C23	118.74 (16)
N2—C8—C21	118.56 (13)	C27—C22—C21	123.32 (15)
N3—C9—N2	113.47 (14)	C23—C22—C21	117.92 (14)
N3—C9—C18	129.35 (14)	C24—C23—C22	118.74 (15)
N2—C9—C18	117.18 (15)	C24—C23—C7	122.71 (15)
N3—C10—C15	129.18 (15)	C22—C23—C7	118.51 (16)
N3—C10—C11	111.38 (15)	C25—C24—C23	120.91 (16)
C15—C10—C11	119.44 (15)	C25—C24—H24A	119.5
C12—C11—C10	121.67 (16)	C23—C24—H24A	119.5
C12—C11—N2	133.34 (16)	C24—C25—C26	120.03 (17)
C10—C11—N2	104.97 (13)	C24—C25—H25A	120.0
C13—C12—C11	117.98 (16)	C26—C25—H25A	120.0
C13—C12—H12A	121.0	C27—C26—C25	120.95 (17)
C11—C12—H12A	121.0	C27—C26—H26A	119.5
C12—C13—C14	121.22 (15)	C25—C26—H26A	119.5
C12—C13—C17	118.67 (17)	C26—C27—C22	120.63 (16)
C14—C13—C17	120.11 (17)	C26—C27—H27A	119.7
C15—C14—C13	119.97 (17)	C22—C27—H27A	119.7
			
C6—C1—C2—C3	−0.6 (3)	N2—C11—C12—C13	−178.08 (17)
Cl1—C1—C2—C3	180.00 (14)	C11—C12—C13—C14	−0.2 (3)
C6—C1—C2—Cl2	178.11 (14)	C11—C12—C13—C17	179.24 (17)
Cl1—C1—C2—Cl2	−1.3 (2)	C12—C13—C14—C15	0.0 (3)
C1—C2—C3—C4	0.1 (3)	C17—C13—C14—C15	−179.43 (18)
Cl2—C2—C3—C4	−178.65 (15)	C12—C13—C14—C16	179.13 (16)
C2—C3—C4—C5	1.0 (3)	C17—C13—C14—C16	−0.3 (3)
C3—C4—C5—C6	−1.5 (3)	C13—C14—C15—C10	0.2 (3)
C2—C1—C6—C5	0.1 (3)	C16—C14—C15—C10	−178.96 (15)
Cl1—C1—C6—C5	179.47 (14)	N3—C10—C15—C14	178.80 (16)
C2—C1—C6—C7	−179.36 (16)	C11—C10—C15—C14	−0.2 (2)
Cl1—C1—C6—C7	0.0 (2)	N3—C9—C18—C20	178.47 (16)
C4—C5—C6—C1	1.0 (3)	N2—C9—C18—C20	−1.0 (2)
C4—C5—C6—C7	−179.58 (17)	N3—C9—C18—C19	1.2 (3)
C8—N1—C7—C23	1.9 (2)	N2—C9—C18—C19	−178.29 (15)
C8—N1—C7—C6	−178.42 (14)	C9—C18—C20—C21	0.5 (2)
C1—C6—C7—N1	−67.7 (2)	C19—C18—C20—C21	177.75 (15)
C5—C6—C7—N1	112.84 (18)	N1—C8—C21—C20	179.41 (15)
C1—C6—C7—C23	111.97 (19)	N2—C8—C21—C20	−0.9 (2)
C5—C6—C7—C23	−67.5 (2)	N1—C8—C21—C22	−2.0 (2)
C7—N1—C8—N2	−179.53 (14)	N2—C8—C21—C22	177.66 (13)
C7—N1—C8—C21	0.2 (2)	C18—C20—C21—C8	0.4 (2)
C11—N2—C8—N1	2.2 (2)	C18—C20—C21—C22	−177.98 (15)
C9—N2—C8—N1	−179.90 (14)	C8—C21—C22—C27	−176.46 (15)
C11—N2—C8—C21	−177.54 (15)	C20—C21—C22—C27	2.0 (2)
C9—N2—C8—C21	0.4 (2)	C8—C21—C22—C23	1.8 (2)
C10—N3—C9—N2	0.31 (18)	C20—C21—C22—C23	−179.73 (15)
C10—N3—C9—C18	−179.18 (16)	C27—C22—C23—C24	0.4 (2)
C8—N2—C9—N3	−178.98 (14)	C21—C22—C23—C24	−177.92 (14)
C11—N2—C9—N3	−0.62 (18)	C27—C22—C23—C7	178.34 (15)
C8—N2—C9—C18	0.6 (2)	C21—C22—C23—C7	0.0 (2)
C11—N2—C9—C18	178.94 (14)	N1—C7—C23—C24	175.85 (15)
C9—N3—C10—C15	−178.96 (17)	C6—C7—C23—C24	−3.8 (2)
C9—N3—C10—C11	0.12 (18)	N1—C7—C23—C22	−2.0 (2)
N3—C10—C11—C12	−179.17 (15)	C6—C7—C23—C22	178.35 (15)
C15—C10—C11—C12	0.0 (2)	C22—C23—C24—C25	0.4 (2)
N3—C10—C11—N2	−0.49 (17)	C7—C23—C24—C25	−177.44 (16)
C15—C10—C11—N2	178.69 (14)	C23—C24—C25—C26	−0.7 (3)
C8—N2—C11—C12	−2.7 (3)	C24—C25—C26—C27	0.3 (3)
C9—N2—C11—C12	179.09 (18)	C25—C26—C27—C22	0.5 (3)
C8—N2—C11—C10	178.81 (15)	C23—C22—C27—C26	−0.9 (2)
C9—N2—C11—C10	0.63 (16)	C21—C22—C27—C26	177.38 (15)
C10—C11—C12—C13	0.2 (2)		
